# Vagus nerve stimulation: a pre-hospital case report

**DOI:** 10.29045/14784726.2020.09.5.2.34

**Published:** 2020-09-01

**Authors:** Stian A. Mohrsen

**Affiliations:** University Hospital Southampton NHS Foundation Trust

## Abstract

**Introduction:**

Vagus nerve stimulation (VNS) is an adjunct therapy to anti-epileptics in patients where combination drug therapy alone has failed. The VNS device resembles an implantable defibrillator, and can be found underneath the clavicle on either side of the chest. By using a strong ring magnet, the device can be manipulated to seize function or operate on higher intensities, depending on how it is applied. The use of vagal stimulation is increasingly common and VNS is being explored for a range of other medical complaints.

**Case:**

This case study discusses the encounter between a paramedic and a woman presenting with a choking sensation, isolated uvular deviation and stable cardiorespiratory functions. Following a short period of observation without adverse events, she was discharged on scene and advised to see her specialist epilepsy nurse.

**Conclusion:**

Side effects of VNS increase with intensity of stimulation and can manifest throughout any branch of the vagus nerve. Its therapeutic mechanism of action is yet to be fully understood. The symptoms of over-stimulation are often frightening but benign, and although life-threatening events are rare, they require rapid recognition and immediate intervention.

## Introduction

Vagus nerve stimulation (VNS) is a palliative adjunct therapy used in some of the 280,000 to 320,000 people suffering from epilepsy in the United Kingdom (UK) (Booth & Thompson, 2010; Scottish Intercollegiate Guidelines Network (SIGN), 2015). VNS is considered when seizure absence is unsuccessful after two appropriate drug therapies have been tried, and in those where curative resection of the brain is not suitable or has failed (Dalic & Cook, 2016; Ghaemi et al., 2010; National Institute of Health and Care Excellence, 2012; SIGN, 2015). VNS is also used together with other anti-epileptic interventions such as callostomy (separating the corpus callosum) and deep brain stimulation. Vagus stimulation therapy is a recognised therapy for epilepsy but it is also being researched for use in a range of other conditions within mental and somatic healthcare (Vale et al., 2011; Yuan & Silberstein, 2015).

## Case presentation

A 39-year-old woman contacted the ambulance service stating that she was choking. On assessment by the attending paramedic, she was sat upright on the sofa, appeared systemically well but was distressed. She had a patent airway, although her uvula was deviated to the left. She had a hoarse voice and was unable to talk properly. She confirmed that she had a sensation of choking, but had good bilateral air entry on examination with no additional sounds. Her physiological observations were all normal and a 3-lead electrocardiogram showed a sinus rhythm of 70 beats per minute.

On further assessment, an implanted device under the patient’s left clavicle was discovered, which turned out to be a VNS. As the patient’s condition spontaneously improved, she explained that she had experienced a ‘tingling’ sensation in her throat. She had interpreted this as the onset of a seizure, so had swiped her ring magnet to activate the VNS. However, the ‘tingling’ sensation had become worse, leading to a cycle of further VNS activations with the magnet and worsening of symptoms until she was unable to speak and felt as though she was choking.

The paramedic provided reassurance and advice, and on learning that the patient did not feel confident about using her device, advised her to contact her specialist neurology team the next day for follow-up and further advice. Now understanding that the choking sensation was a common side effect and nothing to worry about, the patient was content with the management plan. The patient was not transported to hospital, so the treating paramedic added that she or anyone around her should still call for help if she had a seizure leading to injury, a seizure lasting more than five minutes or repeat seizures without recovery in between (BMJ Best Practice, 2020).

## Discussion

The tenth cranial nerve consists of a roughly 20/80 percent composition of motor and sensory fibres respectively (Ruffoli et al., 2011). Exiting the brainstem on both sides, trailing down the carotid arteries towards the abdomen it innervates the pharynx, larynx, cardiorespiratory organs and the gastrointestinal tract. Its functions include parasympathetic control of most internal organs, but even more so providing sensory information about the body’s internal environment to the brain, controlling neurological and endocrine responses (Krahl, 2012). The cervical segments are asymmetrical in that the right nerve communicates to the sinoatrial node (reducing heart rate), while the left branch commands the atrioventricular node (suppressing conduction). Exploiting this physiology, VNS makes use of an implantable device similar to an automatic implanted cardioversion defibrillator (AICD).

A VNS presents as a distinctive solid mass in the infraclavicular region on either side depending on which vagal branch it is applied to. This will most often be the left side, as the contralateral branch has been thought to more dominantly affect the cardiorespiratory systems, but Krahl (2012) deems this as dogma stemming from studies in animals with different vagus nerve anatomy to humans. Yuan and Silberstein (2015) support this and affirm right-sided VNS as equally efficacious in arresting seizure activity.

Arrhythmias are not uncommon in implantation of VNS, with an occurrence rate of asystole in the implantation of a left-sided VNS equalling 1 in 1000 (Krahl, 2012). Despite this, no authors have reported on fatal asystole outside of the operating theatre. In similar fashion to AICDs, the vagus stimulator is terminated by *holding* a strong magnet over the device, but can in contrast also be triggered to enhance stimuli by *swiping* a magnet across it, i.e. when a patient perceives an aura – a feature not applicable to defibrillators (Deyell et al., 2010; Fisher et al., 2014; Ramani, 2008).

Vagus stimulation has been found to reduce frequency and intensity of seizures in 50–80% of patients, but its exact mechanism of action is not fully understood (Chrastina et al., 2018; Englot et al., 2011; García-Navarrete et al., 2013). Granbichler et al. (2015) were unable to establish its efficacy in reducing mortality, but linked VNS to an increased risk of premature death as this therapy is dominant in patients with an already volatile and poorly controlled seizure illness. It is not thought that VNS itself affects these numbers either way. A study published by Chrastina et al. (2018) looked at patients followed up after long-term therapy. This found that VNS effectively maintains seizure control, but also that many initial VNS non-responders achieved a reduction of seizures after the two-year follow-up.

Looking at adverse effects of VNS, contrasting low- and high-level stimulation, Panebianco et al. (2015) asserted dysphonia and dyspnoea as the two most common adverse effects. Additional side effects identified include cough, pain, paresthesia, nausea, headache and torticollis (Al Omari et al., 2017). Ardesch et al. (2010) reported vocal cord affection as a negative side effect occurring even at sub-therapeutic amplitude levels. Krahl (2012) later argued that dysphonia and coughing are reproducible in anyone when VNS is applied. This is due to the nerve’s role in controlling the pharynx and larynx, hence why the patient in this case study demonstrated deviation of the uvula on overstimulation. Most literature describes these side effects as benign, albeit bothersome and frightening.

Conversely, Bhatt et al. (2010) report on a presentation of a threatened airway following seizures and incrementally stronger vagus stimulation. As parasympathetic stimuli increase, so does the risk of unwanted cholinergic upper respiratory effects such as glottal and vocal cord dysfunction, or lower respiratory effects such as bronchoconstriction. Adverse cardiovascular effects can take the form of bradycardia or asystole, and their significance cannot be overstated despite a low number of documented cases (Cantarín-Extremera et al., 2016; Fleisher et al., 2011). A case study by Iriarte et al. (2009) reports on a woman experiencing asystolic syncopes as a result of her VNS. This case accentuates the importance of accurate questioning and investigations to differentiate between seizures and cardiac syncopes in this patient group. The paramedic must also judiciously differentiate expected side effects from potentially life-threatening events, and be prepared to intervene urgently.

Vagus stimulation is also used in mood disorders and is being considered for application in an array of other conditions; therefore, it is reasonable to assume that paramedics are increasingly likely to encounter these patients in the community. The management of these patients is not clearly defined in the literature. Treatment and therapies must be based on knowledge on pathophysiology and pharmacology and translated from existing guidelines for treating the presenting symptoms. In a patient presenting with a threatened airway, oxygen therapy in conjunction with bronchodilators and non-invasive ventilation proved efficacious in palliation of adverse effects in one subject, but it was ultimately termination of the vagus stimulation that restored airway stability (Bhatt et al., 2010). In the absence of recommendations on managing these patients in extremis, it is felt that early consideration for contacting senior medical advice to optimise management of these patients is necessary.

Little literature exists on severe adverse events, and the vast majority of side effects are provoking or frightening but nevertheless benign. Underlying knowledge about the autonomic nervous system, cranial nerve examination, correlating pathophysiology and VNS therapy can equip the paramedic to provide reassurance and advice, as well as managing a patient presenting in extremis. Epileptics receiving VNS will generally be allocated to a neurology specialist or team that can provide advice or an opportunity for onwards referral and follow-up. It could be argued that any side effect significant enough to cause 999 activation should as a bare minimum be communicated to the specialist team to flag up the event. Clearer guidance on the management of those with a VNS and adverse signs and symptoms is currently lacking but could enable paramedics to make confident management plans when encountering this rare patient group.

## ‘Take-home’ messages

- The number of people living with epileptic VNS therapy is increasing
- VNS is also used in people with mood disorders, and has a potential role in a range of other pathologies
- Most side effects of VNS are bothersome but do not require immediate intervention
- Life-threatening adverse events such as severe asthma-like symptoms, or bradycardia or asystole can occur, albeit very rarely
- Successful therapy appears to rely on identification of an implanted VNS device, discontinuation of stimuli and symptomatic treatment of cholinergic effects

## Acknowledgements

I want to thank Dr Matt Kerton for his help in finding an expert who could inform me where evidence on resuscitation of those with vagus nerve stimulators is absent or unclear.

## Guarantor statement

SAM acts as the guarantor for this article.

## Conflict of interest

None declared.

## Disclaimer

Patient information has been anonymised to comply with current UK legislation, and informed consent to publish this case study was obtained from the patient.

## Funding

None.
